# Crossed Leg Sign Is Associated With Severity of Unilateral Spatial Neglect After Stroke

**DOI:** 10.3389/fneur.2018.00256

**Published:** 2018-04-18

**Authors:** Gustavo José Luvizutto, Eduardo de Moura Neto, Luiz Antônio de Lima Resende, Hélio Rubens de Carvalho Nunes, Luiz Eduardo Gomes Garcia Betting, Rodrigo Bazan

**Affiliations:** ^1^Department of Applied Physical Therapy, Institute of Health Sciences, Federal University of Triângulo Mineiro (UFTM), Uberaba, Brazil; ^2^Department of Physical Therapy, Faculty of Human Talent (FACTHUS), Uberaba, Brazil; ^3^Department of Neurology, Psychology and Psychiatry, Botucatu School of Medicine, University Estadual Paulista Júlio de Mesquita Filho (UNESP), Botucatu, Brazil; ^4^Department of Public Health, Botucatu School of Medicine, University Estadual Paulista Júlio de Mesquita Filho (UNESP), Botucatu, Brazil

**Keywords:** crossed leg sign, unilateral spatial neglect, hemispatial neglect, stroke, cerebrovascular disease

## Abstract

**Background:**

The crossed leg sign in patients with right hemisphere stroke is thought to be associated with perceptual disorders, such as unilateral spatial neglect (USN). The aim of this study was to compare the crossed leg sign with the severity of USN during the acute phase of stroke.

**Experimental procedures:**

This was an observational and prospective clinical study of individuals with a diagnosis of right parietal stroke, as confirmed by neuroimaging. The occurrence of the crossed leg sign, the time at which this occurred after the stroke, and a clinical diagnosis of USN were measured and recorded. The patients’ age, sex, and lesion severity, as determined by the National Institutes of Health Stroke Scale and Glasgow coma scale, were included in the analyses as confounding variables. The outcome of interest was the degree of USN, as measured by the cancellation and bisection tests. Binary logistic regression was used to analyze the effect of crossed leg syndrome on the severity of USN. In the adjusted multiple regression model, a *p*-value of <0.05 was considered statistically significant.

**Results:**

Overall, 60 patients were included in this study. There were no associations between patient demographics and the presence of the crossed leg sign. There was, however, an association between the crossed leg sign and the absolute value of the deviation in the line bisection test (*B* = −0.234; *p* = 0.039). The crossed leg sign was not associated with other measures of USN.

**Conclusion:**

Based on the results of our study, we can conclude that a crossed leg sign in the acute phase of stroke is associated with USN severity, specifically the misinterpretation of the midline.

## Introduction

Unilateral spatial neglect (USN) is a perceptual disorder that is characterized by an inability to respond to people or objects that are presented contralateral to the lesioned side of the brain when these symptoms cannot be attributed to either motor or sensory deficits (1–3). USN is frequently demonstrated in the clinic as misinterpretation of the midline, which may include head and eye deviations on the side contralateral to hemiplegia as well as the crossed leg sign (4–6).

The crossed leg sign was first described in patients with right hemisphere stroke who presented with USN, including cases in which there were associated changes in consciousness (7). It is impossible to detect USN during coma, but frequent rubbing movements of the right leg over the left observed in the first days of clinical evolution may differentiate between patients with torpor and coma. This sign is characterized by an overlap of the right leg over the left as the patient attempts to orient to the midline because there is a loss of spatial orientation of the left space. If the left leg is not perceived or felt to be one’s own limb, then abnormal rubbing movements may appear, which may be of predictive value in the development of USN (7).

Our hypothesis is that patients with the crossed leg sign may have a perceptual disorder that causes severe USN after stroke. The aim of this study was to compare the crossed leg sign with the severity of USN in the acute phase of stroke.

## Materials and Methods

### Study Design, Setting, and Participants

This was a prospective clinical study in individuals with a diagnosis of stroke that had been confirmed by computed tomography or magnetic resonance imaging. Patients hospitalized in the Emergency Room at the Stroke Unit at the Botucatu Medical School in Botucatu, Brazil were included in this study and were followed from January to December 2016. Stroke diagnoses were established according to the routine guidelines of the hospital. In the hyperacute phase (up to 8 h following the stroke), the CT scan is performed without contrast and extra and intracranial angiotomography, in addition to perfusion CT. In patients in the acute phase (after 8 h following the stroke), the CT scan is performed without contrast and extra and intracranial angiotomography. In all subjects, unconfined CT is repeated to obtain a control. Contrast magnetic resonance imaging (T1, T2, flair, and diffusion) is only performed to exclude stroke mimics, neuroinfections, and tumors.

### Inclusion and Exclusion Criteria

Male and female subjects aged 18–85 years with right parietal ischemic stroke confirmed by a CT scan or MRI in the acute phase were included in this study. Individuals with a prior modified Rankin Scale >1, aphasia, preexisting dementia, previous visual changes, associated hemianopsia, mechanical orthopedic changes that impair the movement of the lower limb, or other neurological diseases were excluded.

### Variables

#### Exposures

The occurrence of the crossed leg sign, the time at which the sign occurred after the stroke, and a clinical diagnosis of USN were measured and recorded.

#### Potential Confounding Variables

The main confounding variables that could potentially be associated with the outcome included age (older individuals have higher severity of USN after stroke), sex (women have worse outcomes of USN in some studies), severity of stroke according to the National Institutes of Health Stroke Scale (NIHSS) (the severity of stroke was associated with higher degree of USN), and the level of consciousness, as measured by the Glasgow coma scale (GCS) (greater degree of USN is expected in individuals with a lower degree of GCS).

#### Outcomes

The outcome of interest was the degree of USN, as measured by the cancellation and bisection tests.

### Data Sources and Measurement

#### USN Tests

The degree of USN in the acute phase of stroke was measured using three tests. The first test was the line cancellation test, which measures the proportion of lines omitted from a total of 40 lines that are randomly distributed on one sheet of paper (8). The second test was the star cancellation test, which measures the proportion of stars omitted from a total of 56 smaller stars that are associated with distractors (9). The final test was the line bisection test (LBT), where “the patient was asked to mark the middle of each of 18 lines arranged in three columns of six (on the right, center, and left of the page); the degree of neglect was measured as the distance (in mm) between the patient’s mark and the middle of the lines” (10). In all USN tests, the examiner placed the exam sheet in front of the patient at a distance of 60 cm from the glabella to the center of the paper (11, 12).

#### Follow-Up of Crossed Leg Sign

All patients were observed for their crossed leg behavior in the hyperacute phase (in the emergency room), in the acute and sub-acute phases (in the stroke unit) and at hospital discharge. Crossed leg behavior was defined as having occurred when the patient had the compulsion to cross one leg over the other for two or more consecutive days in three periods of the day. The presence or absence of the sign and the length of time that it remained were recorded. For a confirmed crossed leg sign, the patient must have exhibited ≥3 occurrences of crossed legs on ≥2 consecutive days, with a duration of at least 30 s. The sign was checked every 2 h. Each time, the examiners uncrossed the patient’s legs to see if the legs returned to the initial pattern. To establish the time at which the sign disappeared, the absence of the crossed leg sign was considered to be the absence of the compulsion to crossed one leg over the other for two or more consecutive days after the examiners uncrossed the patient’s legs.

The patient’s severity and topography were blinded for and then followed by five different observers with different levels of clinical experience. Two were consultants (Eduardo de Moura Neto and Hélio Rubens de Carvalho Nunes) and two were neurologists (Luiz Antônio de Lima Resende and Luiz Eduardo Gomes Garcia Betting). The first author (Gustavo José Luvizutto) and the last author (Rodrigo Bazan) recorded the responses from the observers.

To avoid selection bias, all patients with right hemisphere stroke were followed during hospitalization for the crossed leg sign.

### Sampling Plan and Determination of the Minimum Number of Subjects

This was a convenience sample, and a minimum of 60 subjects was needed to obtain a maximum sampling error of 7.5% and a confidence level of 95%. The type I and II error probabilities were 0.05 and 0.20, respectively, with a SD of the mean of 20 for the left percentage deviation required to detect a difference equal to 10 points in relation to the mean of the left percentage deviation in the LBT.

### Statistical Analysis

Binary logistic regression was used to analyze the effect of crossed leg syndrome on USN level after adjusting for the potential confounding variables (age, sex, severity of stroke, and GCS). In the adjusted multiple regression model, a *p*-value of <0.05 was considered statistically significant. The proportion of agreement (presence or absence of cross leg sign) was calculated by the kappa coefficient. A kappa coefficient value less than 0.00 suggests poor agreement, 0.00–.20 suggests slight agreement, 0.21–0.40 suggests fair, 0.41–0.60 suggests moderate, 0.61–0.80 suggests substantial, and 0.81–1.00 suggests almost perfect agreement. Statistical analyses were performed with SPSS software version 24.0 (IBM^®^, Chicago, IL, USA).

### Ethical Aspects

This research was approved by an Ethics Committee on Human Research at Botucatu Medical School (number 4223-2012), and written informed consent was obtained from the participants of this study.

## Results

**Figure 1:**
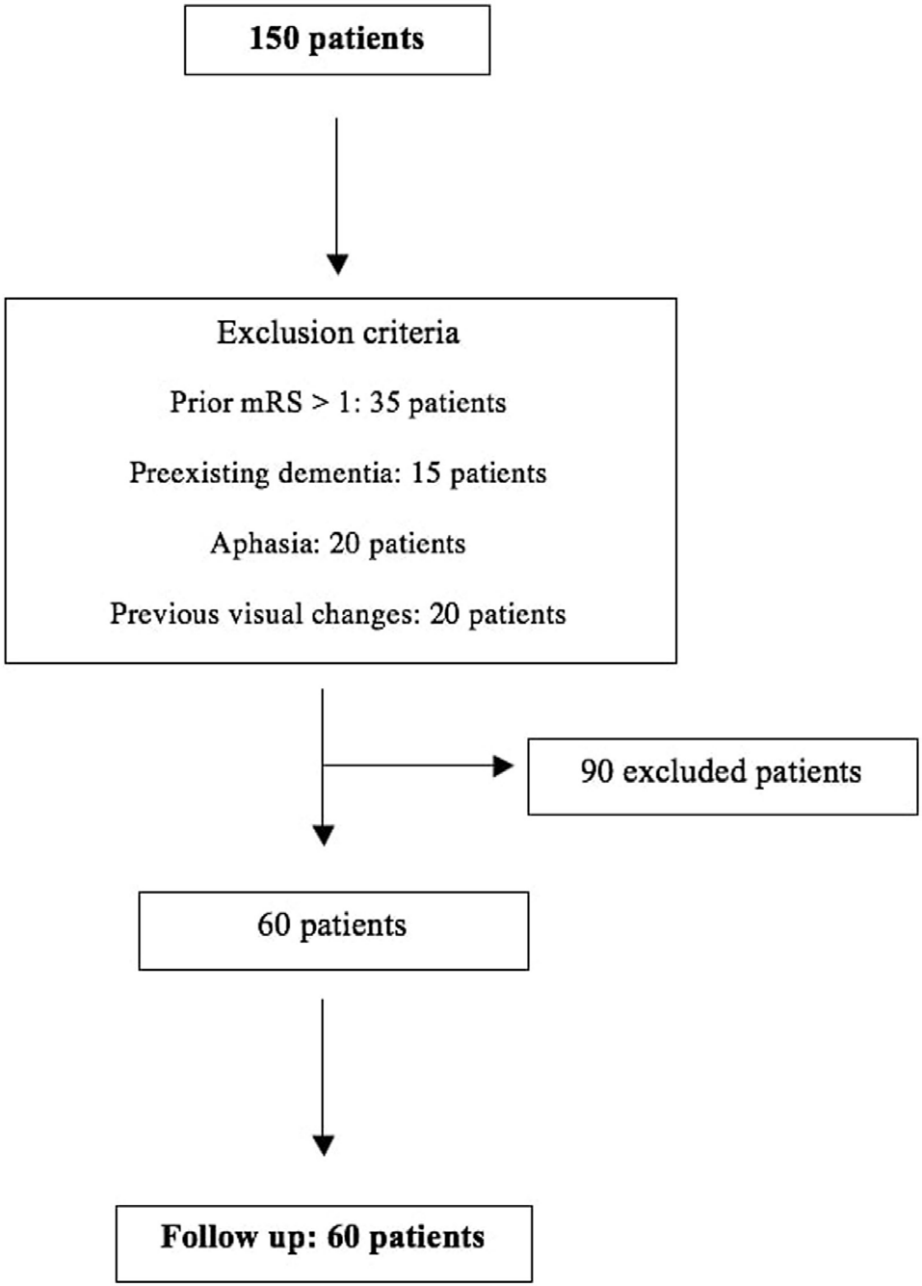
Flowchart.

Overall, 150 patients were screened for this study, and 90 were excluded based on the exclusion criteria. Thus, 60 patients were followed and included in the analyses (Figure 1). The demographic and clinical characteristics are shown in Table 1. The proportion of agreement in the observations of the crossed leg sign between the authors was 86% (74–91%).

**Table 1:** Demographic and clinical characteristics of the patients.

	Min	Max		Mean	SD
Age	34	89		68.6	13.3
Sex					
Male		47 (78.3%)			
Female		23 (21.7%)			
NIHSS	3	24		10.7	5.2
GCS	7	15		12.1	2.7
Cross leg					
Yes		26 (43.3%)			
No		34 (56.7%)			
LCT	0	40		22.5	12.1
SCT	6	52		33.6	12.6
AVD	15.7	98.8		66.4	22.3
NRD	12	19		17.1	2.0

*NIHSS, National Institutes of Health Stroke Scale; LCT, line cancellation test; SCT, star cancellation test; AVD, absolute value of midline deviation; NRD, number of right deviation of the midline; GCS, Glasgow coma scale*.

Table 2 describes the clinical and demographic differences between the patients who did and did not present the crossed leg sign. There was no statistically significant difference between the groups regarding the clinical and demographic profile, only an absolute value of midline deviation (*p* = 0.023).

**Table 2:** Demographic and clinical characteristics of the patients with and without crossed leg sign.

Variables	CL+ (*n* = 26)	CL− (*n* = 34)	*p* Value
Age	68 (34–89)	65 (36–82)	0.111
Male	21 (80.7%)	24 (70.6%)	0.074
Female	5 (19.3%)	10 (29.4%)	0.091
NIHSS	10 (3–24)	10 (5–20)	0.594
GCS	12 (7–14)	12 (7–15)	0.452
LCT	22 (0–40)	18 (0–40)	0.294
SCT	33 (6–52)	30 (6–48)	0.535
AVD	66 (15–97)	43 (5–32)	0.023
NRD	18 (14–19)	16 (12–19)	0.192

*CL+, crossed leg sign positive; CL−, crossed leg sign negative; NIHSS, National Institutes of Health Stroke Scale; LCT, line cancellation test; SCT, star cancellation test; AVD, absolute value of midline deviation; NRD, number of right deviation of the midline; GCS, Glasgow coma scale*.

**Figure 2:**
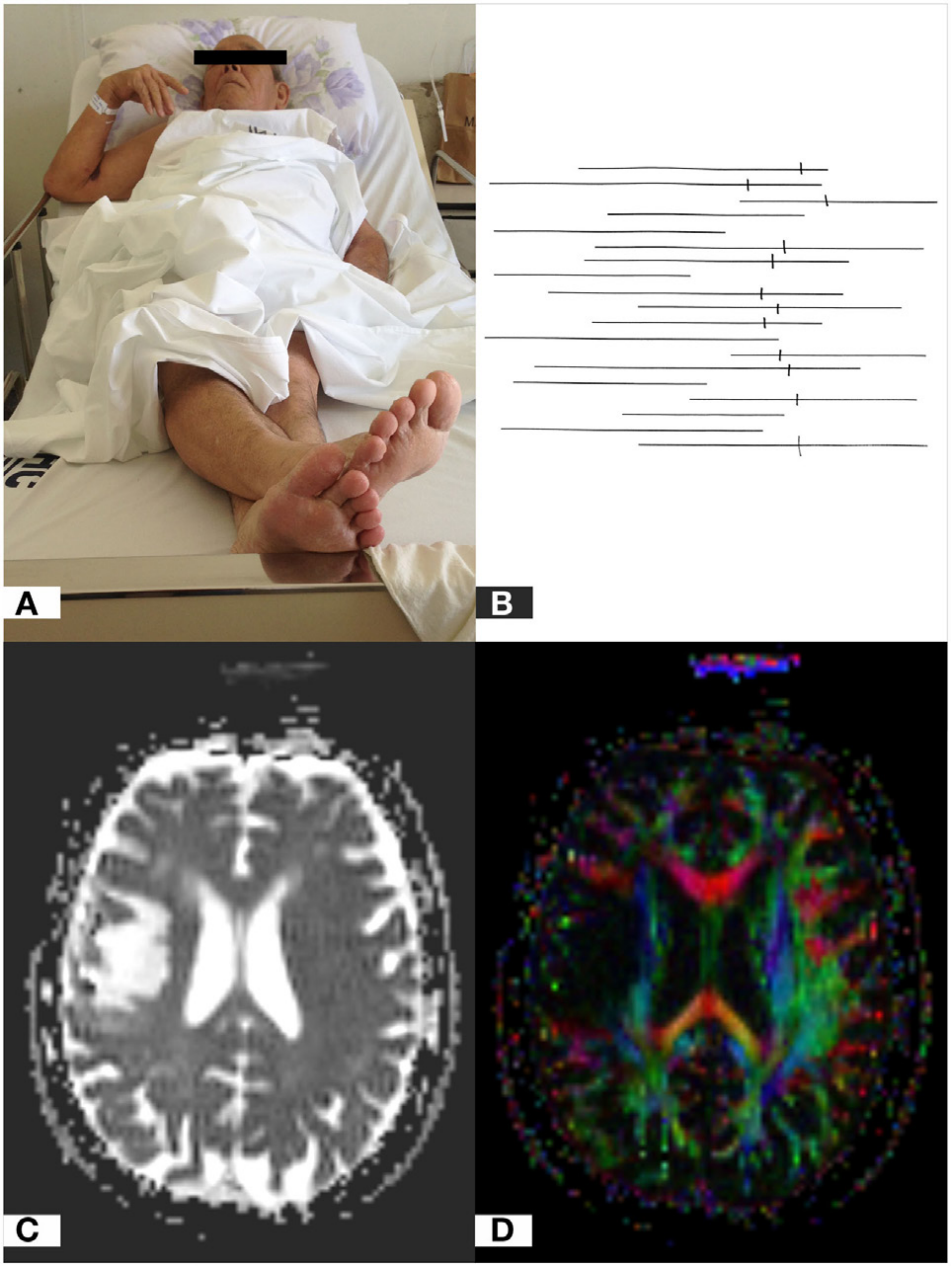
Crossed leg sign in a patient with unilateral spatial neglect **(A)**; line bisection test showing right deviation of the midline **(B)**; axial apparent diffusion coefficient map **(C)**; and diffusion tensor imaging tractography **(D)** showing a lesion in the right frontoparietal region involving cortico-subcortical portions.

In Figure 2, we show the crossed leg behavior in a patient (Figure 2A) with an objective diagnosis of USN by the LBT (Figure 2B), and the axial apparent diffusion tensor (Figure 2C) and diffusion tensor imaging tractography (Figure 2D) show a lesion in the right frontoparietal region. In the LBT, the lines to the left on the paper were not marked, being compatible with the objective diagnosis of USN.

There was no relationship between any of the patient demographics and the presence of the crossed leg sign. There was a relationship between the crossed leg sign and the absolute value of the deviation on the LBT (*B* = −0.234; *p* = 0.039). There were no associations between the crossed leg sign and the other USN tests (Table 3).

**Table 3:** Binary logistic regression according to the crossed leg with USN degree corrected by clinical and anthropometric variables.

Variables	*B*	SE	Wald	*p* Value
Age	−0.128	0.08	2.545	0.111
Sex (male)	−3.856	2.187	3.109	0.078
NIHSS	−0.104	0.196	0.284	0.594
GCS	−0.102	0.089	0.179	0.576
LCT	−0.057	0.145	0.154	0.694
SCT	−0.05	0.081	0.386	0.535
AVD	−0.234	0.113	4.282	0.039
NRD	−0.562	0.429	1.714	0.19
Constant	45.923	19.409	5.598	0.018

*Wald, Wald test; NIHSS, National Institutes of Health Stroke Scale; LCT, line cancellation test; SCT, star cancellation test; AVD, absolute value of midline deviation; NRD, number of right deviation of the midline; GCS, Glasgow coma scale*.

## Discussion

This study indicates that crossed leg behavior is a factor describing the severity of the development of USN after stroke. It has been shown that cross-legged individuals are twice as likely to present right midline deviations in the LBT. This was an important factor in establishing a causal relationship between leg crossing and the severity of USN and misinterpretation of the midline.

Rémi et al. described 34 patients with leg crossing behavior after stroke and reported that the median duration for this behavior was 15 days and was associated with the best functional prognosis. The authors of that study did not, however, correlate this finding with USN (13). No association was found with better outcomes in this study because the patients with USN presented higher NIHSS values, indicating a higher severity of the neurological condition.

Several textbooks on semiology and neurological examination do not describe the crossed leg sign as a clinical sign in the acute phase of stroke (14–16). In recent years, however, patients with acute stroke in the right hemisphere and changes in consciousness have been reported to present with this behavior during hospitalization, and this appears to be especially true in patients with USN and misinterpretation of the midline (7).

The authors postulate three main theories for leg crossing behavior in the acute phase of stroke in patients with USN. The first is that perceptual disorders with a misinterpretation of the midline, such as anosognosia and asomatognosia, are mainly associated with USN with parietal syndrome of the right hemisphere (9, 17). As an individual’s consciousness is altered and he/she perceives a foreign body in the bed, such as his/her left foot or anything in the left hemisphere, the right foot would be expected to repeatedly and compulsively perform the movement of encountering and rejecting this member. The second theory is that when misinterpreting the midline, these patients are always searching for sensorial stimuli. These stimuli increase extrinsic feedback from sensorial input and allow a better interpretation by the parietal lobe (18, 19). The final theory is the inter-hemispheric inhibition theory, which suggests that in the acute phase of stroke, a patient with hemiplegia associated with USN will use the compromised lower limb less frequently, which results in less information being transmitted to the injured parietal cortex (20, 21). As the injured hemisphere receives less information, it will no longer inhibit the uninjured hemisphere, leading to a hyperactivation of the contralesional hemisphere and an increase in motor activity on the unaffected side.

Based on the results presented, we conclude that the crossed leg sign in the acute phase of stroke is associated with severity of USN, specifically in the misinterpretation of the midline.

## Ethics Statement

This research was approved by an Ethics Committee on Human Research at Botucatu Medical School (number 4223-2012).

## Author Contributions

GL—literature search, data collection, and writing; EN—writing; HN—data analysis and interpretation; LR—data interpretation and study design; RB and LB—study design.

## Conflict of Interest Statement

The authors declare that there is no conflict of interest regarding the publication of this article.
